# Urea versus fractional Er:YAG laser pretreatment of methylene blue photodynamic therapy in the treatment of moderate toenail onychomycosis: short- and medium-term effects

**DOI:** 10.1007/s00403-022-02448-7

**Published:** 2022-10-31

**Authors:** Enrique Alberdi, Clara Gómez

**Affiliations:** 1 Private Clinic of Dr. Alberdi, Aviador Zorita 25, 28020 Madrid, Spain; 2 Institute of Physical Chemistry Rocasolano, Spanish National Research Council, CSIC, C/ Serrano 119, 28006 Madrid, Spain

**Keywords:** Fractional Er:YAG laser, Urea, Methylene blue, Onychomycosis, Photodynamic therapy

## Abstract

Keratolytic properties of urea 40% have long time used for the treatment of onychomycosis. Fractional ablative lasers enhance the delivery of topically applied photosensitizers improving photodynamic therapy (PDT) efficacy. The aim of this study was to compare the short- and medium-term efficacy of a pretreatment with urea 40% and fractional Er:YAG (Fr Er:YAG) laser radiation before PDT mediated by methylene blue (MB) for moderate toenail onychomycosis. Twenty-first-toe toenails were randomized to receive either urea 40% (Group I) or Fr Er:YAG laser (Group II) pretreatment and 9 sessions of MB/PDT over the course of 16 weeks. At baseline, 28- and 40-week follow-ups, clinical efficacy was assessed by digital photographs [allowing determination of the onychomycosis severity index (OSI)], whereas mycological efficacy was assessed by histological examination and fungal culture. Details of the side effects and patients’ satisfaction were also recorded. In both groups, a significant decrease in OSI values was observed at the 28-week follow-up and a slight rebound at the 40-week follow-up. The percentage of nail involvement decreased significantly in both groups at the 28-week follow-up, to continue declining gently in Group I at 40 weeks, in contrast to the rebound observed during this period in Group II. The mycological cure rate was 20% and 30% at 28-week follow-up and 70% and 40% at 40-week follow-up, in Group I and II, respectively. Patients reported being fairly satisfied, and no side effects were detected in any groups. Although both pretreatments favor the action of PDT for the treatment of onychomycosis, the use of urea at 40% is more effective in the medium term.

## Introduction

Fractional photothermolysis is considered a significant technological advance in dermatology. In fractional lasers, the laser beam is split into a pattern of microbeams. This results in thermal microscopic wounds into deep skin structures [1]. Fractional lasers can be used as laser-assisted delivery systems since they promote an increase in the contact area with the nail, generation of channels for deeper penetration and disruption of the keratin strands, allowing topical drugs or bioactive compounds to penetrate across the nail plate [2].

Onychomycosis is a fungal nail infection often with thickening, discoloration and separation from the nail bed, being more common in the elderly [3]. Dermatophytes are the most frequent causative agents [mainly Trichophyton rubrum (*T. rubrum*)] along with non-dermatophyte molds and yeasts to a lesser extent. Physical and microscopic examination and culture are needed for an accurate diagnosis. In addition, biopsy of the nail histologically evaluated using periodic acid–Schiff (PAS) staining increases the accuracy for detecting infection [4]. Oral and topical antifungals are the most commonly available options for treatment for onychomycosis, showing some disadvantages as important adverse reactions and drug interaction (oral agents) and poor efficacy due to their poor permeability (topical agents) [5].

In view of the limited efficacy of traditional therapies along with its high prevalence, it is needed to explore new and more effective alternative therapies in treating the disease. Lasers are an innovative technology for the treatment of moderate onychomycosis [6]. Although it is not clarified yet, the therapeutic effect of the laser radiation can be generated by direct inactivation of fungi by heat or selective photothermolysis, even though a target chromophore has yet to be identified [7]. Photodynamic therapy PDT has been revealed as a promising procedure for onychomycosis treatment by reactive oxygen species [8]. To improve the spread and delivery of topically applied photosensitizers across the nail to enhance the healing effects of PDT and reduce recurrence, a pretreatment of thick lesions is recommended [9]. A short while ago, the increase in the PDT efficacy via combination with ablative lasers has begun to be studied [10, 11]. Ablative laser promotes topical photosensitizer delivery to the nail plate, but more systematic studies are needed to establish an appropriate protocol [12].

Moreover, studies involving the use of urea as a therapeutic option for onychomycosis have been newly reviewed [13]. This review suggests that available studies have not proved that urea by itself is any better than standard treatments, but that it is a good adjunctive treatment to improve the efficacy of those [13].

Based in these considerations, a controlled short- and medium-term controlled clinical trial is proposed to assess and compare the efficacy of a pretreatment with urea with another pretreatment based in fractional Er:YAG laser (Fr Er:YAG) radiation before PDT mediated by methylene blue (MB/PDT) for moderate toenail onychomycosis.

## Materials and methods

### Study design

**Fig. 1:**
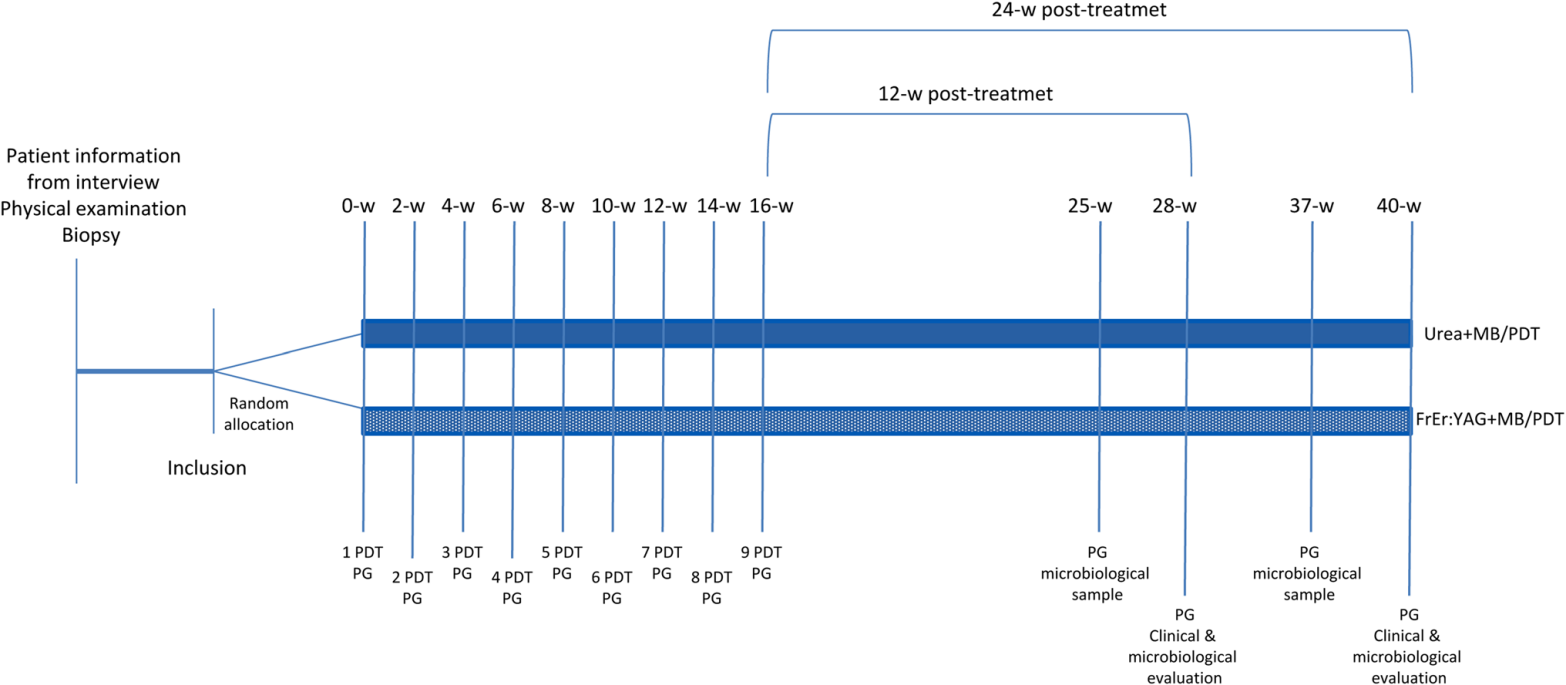
Graphical representation of the chronological order of the experimental interventions and clinical and microbiological assessment (w, week; PG, photograph)

A randomized controlled trial of 40-week duration was designed (Fig. 1). The study included patients with moderate (values for onychomycosis severity index (OSI) between 6.0 and 15.9) first-toe toenail onychomycosis, with mycological diagnosis of distal and lateral subungual onychomycosis (DLSO) by histological examination of nail clipping using PAS staining and fungal culture. These patients were recruited from a private dermatology clinic and divided randomly into two groups: Group (I) included 10 nails pretreated with 40% urea and treated with 9 sessions of MB mediated PDT (MB/PDT) and Group (II) included 10 nails treated with 9 sessions of Fr Er:YAG laser combined with MB/PDT, in both cases, over the course of 16 weeks. The inclusion in one group or another was done randomly using online computer software (Research Randomizer). Demographic characteristics of recruited patients and basal clinical parameters of the affected first-toe toenails are shown in Table 1.

**Table 1:** Demographic data of recruited patients and basal conditions of the affected first-toe toenails

	Urea + MB/PDT	Fr Er:YAG + MB/PDT
Age		
[mean ± (SD)]; range; median	62.0 ± 14.4; [45,81]; 57	53.6 ± 14.5; [33,78]; 61
Sex		
Male [*n* (%)]	4 (40%)	4 (40%)
Female [*n* (%)]	6 (60%)	6 (60%)
Culture reports [*n* (%)]		
T rubrum	10 (100%)	8 (80%)
T interdigitale	0 (0%)	2 (20%)
OSI [mean ± (SD)]	12.2 ± 5.3	15.9 ± 6.1
Nail involvement (%) [mean ± (SD)]	38.0 ± 20.8	31.5 ± 21.5
Chronicity (years) [mean ± (SD)]	1.9 ± 1.0	2.7 ± 1.3

*n* = number of patients; (%) = percentage of patients; SD = standard deviation

Exclusion criteria were: under 18 years of age, photosensitive disorders, deficiency of glucose-6-phosphate dehydrogenase, concurrent nail diseases: psoriasis and lichen planus, internal diseases, immunosupression, pregnancy, tinea pedis, use of topical or systemic antimycotic agents within 0–6 months before enrollment and physical or laser treatment modalities for at least 6 months before participation in the study [14].

The ethical approval was obtained from Ethics Committee of the Hospital Clínico San Carlos, Madrid, Spain (Internal code: 17/501-E) in compliance with the Declaration of Helsinki (2013) [15]. An informed consent form was signed by each patient before their inclusion in the experimental trial.

### Pretreatment

#### Group I (urea 40% pretreatment)

40% urea ointment was employed to soften the plates of the affected nails and vaseline was applied in periungual skin to prevent irritating effects of urea at high concentrations. Once the urea and vaseline were applied, the nail was covered by an occlusive dressing for 12 h at night. Urea softening treatment was limited to 4–5 days in toenails with hyperkeratosis < 2 mm and 7 days in those with hyperkeratosis ≥ 2 mm [16].

#### Group II (Fr Er:YAG pretreatment)

**Fig. 2:**
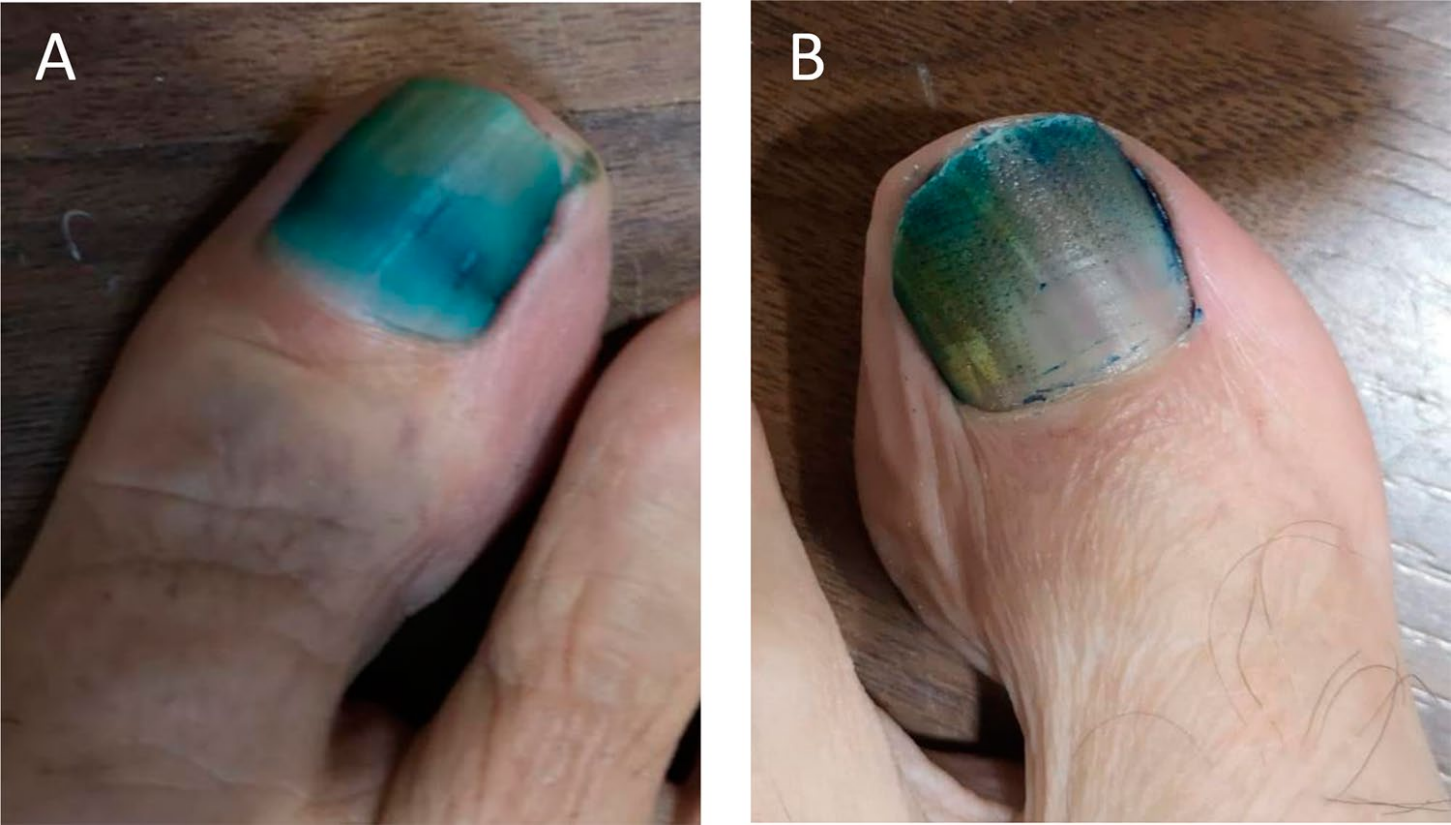
**A** Nail pretreated with urea and **B** with Fr Er:YAG before receiving fifth MB/PDT session

Fractional ablative treatment was carried out using the Pixel^®^ 2940 nm Module of the Harmony platform (Alma Lasers Ltd., Caesarea, Israel) which incorporates a microlens aligned in a matrix of 9 × 9 (81) dots (pixels), emitting 17 mJ per pixel when using the maximum pulse energy output of 1,400 mJ. The single-pass ablation microzone is around 150 µm in diameter per pixel. During laser application, the handpiece was kept static, and that is why the laser beams always reached the same points creating holes on the nail plate surface. Nine pulses per area treated were applied scanning the entire affected surface (Fig. 2).

### Treatment

After any of the pretreatment used, a PDT mediated by MB (MB/PDT) was carried out, for which a solution of MB in water (2%) was applied to the affected nail, and 3 min later, the nail was photoactivated by a red light-emitting diode (LED) lamp (Aktilite^®^, Photocure ASA, Oslo, Norway) (λ = 635 nm, fluence 37 J/cm^2^, irradiance 70 mW/cm^2^, exhibition time 10 min). A fixed distance of 100 mm was always maintained between the lamp and the affected nail. PDT was repeated nine times with 2 weeks in between.

### Monitoring and assessment timeline

Clinical efficacy was assessed by digital photographs recorded at baseline, each PDT sessions and follow-up visits which allow monitor the severity and the evolution of the disease, in accordance with the onychomycosis severity index (OSI) obtained [17]. To calculate the OSI value, the area of involvement, the proximity of the disease to the matrix, the presence of longitudinal streaks, patches and subungual hyperkeratosis greater than 2 mm were taken into account [17].

The degree of improvement was calculated as the decrease in the percentage of OSI score at baseline and follow-ups. The ranges of values used to classify that degree of improvement were: 0–25% (no improvement), 26–50% (mild improvement), 51–75% (moderate improvement) and 76–100% (outstanding improvement) (Table 2) [18].

**Table 2:** Comparison of clinical and mycological response and patients’ satisfaction between groups over the period evaluated

	Urea + MB/PDT	Fr Er:YAG + MB/PDT
OSI [mean ± (SD)]		
Baseline	12.2 ± 5.3	15.9 ± 6.1
28-w follow-up	2.7 ± 1.8^a^	7.1 ± 6.2^a^
40-w follow-up	3.7 ± 3.6^a^	8.5 ± 5.6^a^
Intergroup *p* value n.s. (> .05)		
Degree of improvement at 28-w follow-up [*n* (%)]		
No (0–25%)	0 (0%)	3 (30%)
Mild (26–50%)	1 (10%)	1 (10%)
Moderate (51–75%)	5 (50%)	1 (10%)
Outstanding (76–100%)	4 (40%)	5 (50%)
Mean value	76.5 ± 17.1	57.2 ± 37.1
Intergroup *p* value n.s. (> .05)		
Degree of improvement at 40-w follow-up [*n* (%)]		
No (0–25%)	2 (20%)	3 (30%)
Mild (26–50%)	0 (0%)	4 (40%)
Moderate (51–75%)	2 (20%)	0 (0%)
Outstanding (76–100%)	6 (60%)	3 (30%)
Mean value	75.3 ± 33.4	44.4 ± 36.2
Intergroup *p* value n.s. (> .05)		
Nail involvement (%) [mean ± (SD)]		
Baseline	38.0 ± 20.8	31.5 ± 21.5
28-w follow-up	11.5 ± 8.5^a^	10.5 ± 8.6^a^
40-w follow-up	4.5 ± 4.0^a^	13.5 ± 8.8^a^
Intergroup *p* value n.s. (> .05)		
Histological analysis*		
Baseline		
PAS stain (+)	10 (100%)	10 (100%)
PAS stain (−)	0 (0%)	0 (0%)
28-w follow-up		
PAS stain (+)	8 (80%)	7 (70%)
PAS stain (−)	2 (20%)	3 (30%)
40-w follow-up		
PAS stain (+)	3 (30%)^a^	6 (60%)
PAS stain (−)	7 (70%)^a^	4 (40%)
Intergroup *p* value n.s. (> .05)		
Patients’ satisfaction [*n* (%)]		
Very dissatisfied	0 (0%)	0 (0%)
Dissatisfied	1 (10%)	0 (0%)
Fairly satisfied	6 (60%)	3 (30%)
Very satisfied	3 (30%)	5 (50%)
Extremely satisfied	0 (0%)	2 (10%)
Mean value	3.2	3.9
Intergroup *p* value n.s. (> .05)		

*n* = number of patients; (%) = percentage of patients; SD = standard deviation

*Fungal culture results showed a good accordance with the findings by histological visualization

n.s. = not significant

a = significance of *intragroup* differences compared to baseline

Mycological efficacy was assessed at baseline, 28-week and 40-week follow-up, by fungal culture of toenail scraps and the biopsy of the own toenail.

Details of side reactions and participants’ satisfaction were recorded at each study visit. To quantify patients’ satisfaction, once the treatment was finished, they were asked to rate their degree of satisfaction on a scale of 1–5: 1 very dissatisfied, 2 dissatisfied, 3 fairly satisfied, 4 very satisfied and 5 extremely satisfied.

### Statistical analysis

Sample size calculation was based on detecting differences of more than 10 points between groups in the primary outcome (Onychomycosis Severity Index, OSI), assuming standard deviation of 6, error of 0.05 and power of 90% [14]. In this way, by using IBM SPSS SamplePower (v3.0) software, it was determined that a sample of 20 first-toe toenails at a 1:1 allocation ratio of urea + MB/PDT to Fr Er:YAG laser + MB/PDT (10 first-toe toenails per group) was required.

To check assumptions of normality for the tested variables (OSI, degree of improvement, % of involvement, mycological cure rate and patients’ satisfaction), Shapiro–Wilk test was used. A two-factor [time (within-subject factor) and treatment (between-subject factor)] repeated measures ANOVA was used with the post hoc Bonferroni correction for multiple comparisons. SPSS software for Windows (version 25; SPSS, Chicago, III) was used for the statistical evaluation of the results. A *p* value of < 0.05 was considered as statistically significant.

## Results

No side reactions were found during the study period. MB application resulted in a widespread temporary discoloration of the toenails (Fig. 2). Both pretreatments were well tolerated and suited with later MB/PDT treatment. Results obtained demonstrated clinical and mycological improvement of the target nails. In the group pretreated with urea, improvement was mainly reached at the end of the evaluated period (40 weeks from baseline), whereas in the group pretreated with Fr Er:YAG laser, clinical improvement was obtained earlier, at 28 weeks from baseline, gradually worsening in the following 12 weeks.

Both groups demonstrated a continuous and significant decline in the OSI scores throughout the first 12 weeks of the evaluated period, but a slight rebound through the following 12 weeks (Table 2). No statistically significant differences were detected between the groups.

Mean degree of improvement at 28 weeks was somewhat higher in the group treated with urea, which remained throughout the following 12 weeks. However, in the group pretreated with Fr Er:YAG laser, the mean degree of improvement decreased in the last 12 weeks evaluated (Table 2).

The percentage of nail involvement decreased significantly in both groups at 28 weeks, declining gently at 40 weeks in the group pretreated with urea, in contrast to the group pretreated with Fr Er:YAG laser, in which it was observed a clear rebound (Table 2).

Despite the different trend of the results showed for each group in the last 12 weeks evaluated, no statistically significant differences were detected between the groups for mean degree of improvement and percentage of nail affectation values at 28 and 40 weeks.

Mycological cure rate was 20% and clinical cure rate was 40% (values of outstanding degree of improvement) at 28 weeks in the group pretreated with urea, increasing significantly up to 70% and 60%, respectively, at 40 weeks (Table 2). As a result, complete cure rate was 20% at 28 weeks and 60% at 40 weeks. In the group pretreated with Fr Er:YAG laser, mycological cure rate was 30% and clinical cure rate was 50% at 28 weeks, and they were 40% and 30%, respectively, at 40 weeks (Table 2). Thus, complete cure rate in group pretreated with Fr Er:YAG laser was 30% at 28 weeks and 40 weeks.

Mean grade of satisfaction was 3.2 for urea pretreatment and 3.9 for Fr Er:YAG laser pretreatment (Table 2). It is more comfortable for the patient to apply an ablative laser, although it may involve some discomfort (mild burning sensation and mild pain) as the number of applied pulses increases, since the patient can disregard the obligation of having to apply urea daily in occlusion. Two patients pretreated with 40% urea showed mild dermal irritation around nail area which did not prevent them from completing the treatment.

## Discussion

The present clinical study was specifically designed to evaluate and compare the effectiveness of two experimental pretreatment options, urea and Fr Er:YAG before MB/PDT for treatment of moderate toenail DLSO.

The main mechanism of Fr Er:YAG laser in combination with MB/PDT is the creation of multiple holes on nail plate to promote the uptake of the MB [19]. Photosensitizer can easily reach subungual hyphae masses and dermatophytomas because the channels reach almost the total thickness of the nail plate. MB permeation to the nail plate is enhanced by generating a larger contact area with the nail. [12]. Generally, the fractional laser treatment used in facial rejuvenation requires less fluence and lower ablation rates than those necessary for the pretreatment of onychomycosis given the lower water content and the greater density of keratin in nails compared to skin. The 2940 nm Er:YAG laser radiation is absorbed by the 10–30% of the constituent water of the nail plate and causes ablation by evaporating the tissue analogously to how it does in human skin. Ablation depth is mainly controlled by laser fluence [19]. The vaporization–exfoliation caused by fractional ablative laser radiation not only aids in fungal removal, but also paves the way for a posterior remodeling of the nail plate that quickly improves its appearance [9].

There are two recent studies where Fr Er:YAG laser has been used in combination with topical antifungal medication, specifically with amorolfine. Morais et al. applied the following settings using Fr Er:YAG laser as pretreatment for onychomycosis: 50 mJ/mtz, 2 ms pulse duration and 1 Hz frequency [12]. Zhang et al. applied 35-60 J/cm^2^ (average fluence 42 J/cm^2^) with a density of 120 spots/cm^2^ per pass over the affected nail plate [19]. In both protocols, the number of pulse shots applied to the same area was established depending on the thickness of the nail plate and patient complaints of pain [19]. Both studies concluded that Fr Er:YAG laser in combination with amorolfine is more effective than topical monotherapy in the short term.

Currently, there is a greater number of studies carried out with Fr CO_2_ laser than with Fr Er:YAG laser, most in combination with topical drugs [18, 20–22]. These studies reported that combination therapy (Fr CO_2_ + topical antifungal agent) had a higher efficacy than Fr CO_2_ laser by itself in the treatment of onychomycosis. Some few studies report the effect of its combination with PDT. In the study reported by Abdallan et al. [10], both MB/PDT and Fr CO_2_ + MB/PDT reduced significantly the OSI scores without significant difference between them, but improvement in nail appearance and patient’s satisfaction were higher in Fr CO_2_ + MB/PDT than PDT by itself. Moreover, de Oliveira et al. showed that Fr CO_2_ laser associated with methyl aminolevulinate (MAL) was effective in patients with onychomycosis of long progression and patients that had undergone previous antifungal therapy, with recurrence and treatment failure [11]. Recently, Arora & Ranjan have proposed an interesting alternative management for onychomycosis that consists of the application of an ablative laser pretreatment after an occlusive pretreatment with 12% urea and finally the topical application of terbinafine. The results look promising [23]. Being a hygroscopic agent, urea makes the nail plate softer and used in occlusion helps provide water chromophore at depth, which is selectively absorbed by laser radiation of CO_2_ or Er:YAG lasers.

The pretreatment of affected nail with 40% urea also favors the penetration of the antifungal agent or the photosensitizer across the nail plate [24, 25]. High concentrations of urea (~ 40%) exert a keratolytic/emollient action causing hydrogen bond breaking and then dissolving keratin [26]. Softening of the nail plate, hyperkeratosis removal and reduction of nail plate thickness are obtained with a pretreatment of 40% urea, allowing an enhanced light penetration through the nail plate and thus improving the clinical response of the PDT by reaching the fungal colonies of the nail bed and nail matrix [27, 28]. While urea concentration, frequency of treatment administration and application times are more or less standardized, there is still no consensus on the application parameters of Fr Er:YAG laser, also because until now there are few studies published with Fr Er:YAG laser since it is an incipient investigation.

Following the criterion widely accepted to consider complete cure: achieve a mycological cure (obtaining negative direct microscopy and culture or PAS negative) and 80% nail appearance improvement as the clinical cure, complete cures were 60% and 30% in the urea and Fr Er:YAG laser pretreatment group, respectively, at 40-week follow-up. This discrepancy in the evolution of the response to treatment between the groups can be explained because the created microchannels by Fr Er:YAG allow the diffusion of the MB, but in the spaces adjacent to the microchannels a lower diffusion rate is expected, and thus, a part of the affected area does not participate in the pretreatment. By using urea 40%, the entire affected area is homogeneously pretreated and for a longer time (12 h during 4–7 days before PDT), which may contribute to a more extensive spread of MB in the affected region and the relapse does not take place within the evaluated period. It is important to point out that despite this, no statistically significant differences were detected between the groups for OSI, degree of improvement and percentage of nail affectation values, probably due to the small sample size recruited to form the groups.

In conclusion, this is a pilot study comparing for the first time, the efficacy of urea 40% and Fr Er:YAG laser as pretreatment for PDT. Further studies are needed to delimit the optimal parameters for ablative and photodynamic process and standardization of treatment protocols. Regarding the application of fractional lasers, it is especially needed to define their power, overlapping or not of the microchannels and frequency of application before PDT or even after maceration of the nail by the urea. Longer follow-up period and longer size sample would be needed to evaluate healing process and the recurrence rate. Urea + MB/PDT and Fr Er:YAG + MB/PDT were both safe and effective in treating and reducing the severity of moderate onychomycosis and a good option as alternative to topical antifungal agents. Although Fr Er:YAG pretreatment augmented patients’ satisfaction without the need of the daily home application of 40% urea cream in occlusion, this last pretreatment was more effective in the medium term.
